# Nurse telephone triage in out-of-hours GP practice: determinants of independent advice and return consultation

**DOI:** 10.1186/1471-2296-7-74

**Published:** 2006-12-12

**Authors:** Eric Peter Moll van Charante, Gerben ter Riet, Sara Drost, Loes van der Linden, Niek S Klazinga, Patrick JE Bindels

**Affiliations:** 1 Department of General Practice, Academic Medical Centre, University of Amsterdam, Meibergdreef 15, 1105 AZ Amsterdam, The Netherlands; 2 Department of Social Medicine, Academic Medical Centre, University of Amsterdam, Meibergdreef 15, 1105 AZ Amsterdam, The Netherlands; 3 Horten Centre, University of Zurich, Bolleystrasse 40, CH-8091 Zurich, Switzerland

## Abstract

**Background:**

Nowadays, nurses play a central role in telephone triage in Dutch out-of-hours primary care. The percentage of calls that is handled through nurse telephone advice alone (NTAA) appears to vary substantially between GP cooperatives. This study aims to explore which determinants are associated with NTAA and with subsequent return consultations to the GP.

**Methods:**

For the ten most frequently presented problems, a two-week follow-up cohort study took place in one cooperative run by 25 GPs and 8 nurses, serving a population of 62,291 people. Random effects logistic regression analysis was used to study the determinants of NTAA and return consultation rates. The effect of NTAA on hospital referral rates was also studied as a proxy for severity of illness.

**Results:**

The mean NTAA rate was 27.5% – ranging from 15.5% to 39.4% for the eight nurses. It was higher during the night (RR 1.63, CI 1.48–1.76) and lower with increasing age (RR 0.96, CI 0.93–0.99, per ten years) or when the patient presented >2 problems (RR 0.65; CI 0.51–0.83). Using cough as reference category, NTAA was highest for earache (RR 1.49; CI 1.18–1.78) and lowest for chest pain (RR 0.18; CI 0.06–0.47). After correction for differences in case mix, significant variation in NTAA between nurses remained (p < 0.001). Return consultations after NTAA were higher after nightly calls (RR 1.23; CI 1.04–1.40). During first return consultations, the hospital referral rate after NTAA was 1.5% versus 3.8% for non-NTAA (difference -2.2%; CI -4.0 to -0.5).

**Conclusion:**

Important inter-nurse variability may indicate differences in perception on tasks and/or differences in skill to handle telephone calls alone. Future research should focus more on modifiable determinants of NTAA rates.

## Background

Over the last decades, the organisation of out-of-hours primary health care in many countries has shifted from practice-based services to large-scale general practitioner (GP) cooperatives [1-3]. These changes were fuelled mainly by an increasing demand for out-of-hours care and the GP's desire to reduce the workload during out-of-hours practice. In recent years, a similar development has taken place in the Netherlands [4]. There are currently more than 130 GP cooperatives in the Netherlands, generally with 40 to 120 full-time participating GPs, which cover over 90% of the entire Dutch population and serve between 50,000 and 500,000 people.

Similar to the UK, out-of-hours triage in the Netherlands is initially performed through telephone contact with nurses who receive, assess and manage incoming calls from patients [5]. The call management options include the provision of information and advice as well as referral to a GP or Accident and Emergency (A&E) service. By and large, telephone nurses decide on the subsequent type of contact, the moment at which a patient's call is passed through to the GP: a telephone call to the patient, a centre consultation, or a home visit. While only very few Dutch GP cooperatives make (experimental) use of computerized telephone advice systems (TAS) [6], nationwide telephone nurses do have access to a broad set of written protocols for the most acute problems, developed by the Dutch College of General Practitioners. During their shift in the out-of-hours centre, GPs are subsequently expected to authorise the content of all telephone contacts handled by the nurses.

Various studies have focussed on the safety and effectiveness of the nurse telephone consultation [5,7,8]. They found a substantial decrease in GP workload without an increase of adverse events, like hospital admissions or deaths. However, within the Netherlands alone, substantial differences in NTAA rates were observed among GP cooperatives, ranging from around 25 to 36 percent [9,10]. Perhaps this indicates a lack of agreement on the precise role of the telephone nurse, or differences in the extent to which nurses made use of the available, previously mentioned protocols [11]. Earlier studies have also reported a substantial variability among nurses both without (US) and with the support of TAS (UK) [12-14]. O'Cathain et al. found that some of the inter-nurse variability was explained by the length of their clinical experience and the type of software used [15]. Overall, little is still known about the determinants that are associated with NTAA. Similarly, it is unknown which determinants are associated with return consultations to the GP *after *NTAA. Such information could prove valuable in the discussion on the professional role and position of the telephone nurse in the triage process during out-of-hours primary care.

We studied the contacts that resulted in an NTAA for the ten most frequently presented problems. Aim of the study was to explore which determinants are related to NTAA (1) or to subsequent return consultations after NTAA (2), and to describe to which extent hospital referral rates are affected by NTAA (3).

## Methods

### Setting

The GP cooperative in the coastal city of IJmuiden participated in the study. Serving a population of 62,291 people with 25 GPs and 8 nurses, it has a well-defined area, variable socio-demographic characteristics, and access to electronic medical records for all GP practices (all contacts in- and out-of-hours). The GP cooperative operates from 5 pm until 8 am from Monday to Friday and 24 hours during the weekends. Apart from 11 pm until 8 am when only one GP is on call, two GPs work alongside, one making home visits and one taking care of centre consultations and telephone calls. They are supported by one nurse, who performs the telephone triage as described before. The service is located in the former Accident and Emergency (A&E) Department of a small district hospital that had to close in 1996 and was subsequently used to harbour the GP cooperative.

### Subjects and data collection

Between 1 November 2002 and 1 March 2003, all incoming calls taken by nurses were registered. Contact information was entered on a specially prepared form. It was completed by the nurses (advice alone) or GPs (all other contacts) and was used to collect demographic data, presented problems (up to a maximum of three), contact managed by nurse or GP, diagnosis (only one, made by GP) and management (nurse or GP). The International Classification of Primary Care (ICPC) was used to code the presented problem(s), diagnoses and management [16]. Prior to this study, all data were anonymised, coded and entered into the computer, using SPSS version 11.5.

In total, 4,902 calls were registered. Next, 2,160 (44.1%) contacts on the ten most frequently presented problems were selected from this database: fever, cough, vomiting, shortness of breath, earache, general abdominal pain, sore throat, lower abdominal pain, headache, and chest pain. Between February and June 2005, retrieval and retrospective data collection of these cases took place from the electronic medical records in IJmuiden. It appeared that 1421/2160 (65.8%) contacts were first presentations, whereas 573/2160 (26.5%) contacts were in fact follow-up contacts of earlier presentations during surgery hours or out-of-hours consultations. Another 166/2160 (7.7%) contacts were excluded due to inaccessibility of records or other reasons, which made it impossible to obtain follow-up data. Also excluded were accidents and injuries, even though they did represent a top-ten problem, but most of these patients showed up without calling the cooperative in advance (38.0%) and passed the telephone nurse by. The 1421 first presentations were made by 1324 patients, 1243 of whom attended the service only once (93.9%).

A follow-up period of two weeks was chosen, because virtually all return consultations that were found during a pilot (n = 351) fell within this period of time (92% within one week). Return consultations were only registered for patients who subsequently contacted a GP for the same problem(s). Information was collected on the time to first return consultation (days) and referral to the hospital (y/n).

### Analysis

Main outcomes in this study were (1) determinants of NTAA during first out-of-hours contact, (2) subsequent return consultations after NTAA, and (3) differences in hospital referral rates at first return consultation after NTAA or GP contact. We used random effects logistic regression analysis with nurses as a random intercept. NTAA (yes/no) was the dependent variable for the first research question. The ten most frequently presented problems were modeled as dummy variables using cough as the reference category. These were kept in the model at all times. The initial set of independent variables at the patient level included sex, age, type of insurance (public or private), social deprivation (y/n, area defined by the local council), time of contact (day and evening versus night), number and type of presented problems, and traveling distance to the GP cooperative. At the nurse level the initial set of independent variables included sex and characteristics of experience: length of clinical experience (defined as 'total number of years worked in jobs for which a nursing qualification was required', dichotomized into <20 years or more); variety of experience (measured by the number of clinical specialties which the nurse had worked in, dichotomized into ≤ 3 or more) [15], and experience in GP practice (yes/no). We did not investigate cross-level interactions, given the limited number of nurses and the lack of convincing theories on mechanisms of action. We made the model more parsimonious by removing non-significant variables, but only if they did not materially (>10%) alter the regression coefficients of significant associations and if the likelihood ratio test [17] indicated a non-significant change in the model's fit (at a two-sided p > 0.05). For the second research question the approach was identical but return consultation after NTAA (yes/no) was the dependent variable. Odds ratios were converted to relative risks (RR) to facilitate interpretation [18]. Confidence intervals were set at the 95% level. All analyses were carried out using Stata statistical software (Release 9.2, Stata Corporation, College Station, TX).

## Results

### Nurse telephone consultations of initial contacts

**Figure 1:**
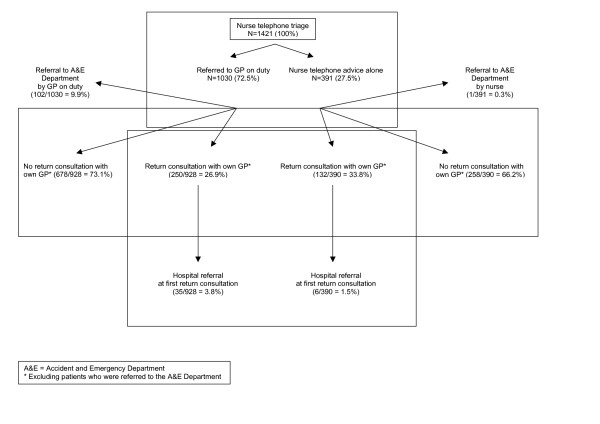
Flow chart of all initial contacts and return consultations.

A flow chart of all initial contacts and return consultations is shown in Figure 1. Out of 1421 calls, 391 (27.5%) were handled by a nurse alone versus 1030 (72.5%) resulting in a GP contact. GPs provided telephone advice (n = 173, 16.8%), centre consultations (n = 675, 65.5%), or home visits (n = 182, 17.7%).

During initial telephone triage, the nurses referred one patient to the A&E services themselves. Another 102 hospital referrals took place via the GP, 2.3% after telephone contact, 6.7% after a centre consultation and 29.1% after a home visit (p < 0.01 for all differences).

Table 1 shows the proportions of calls handled by the nurse alone and proportions of subsequent return consultations (sex, age groups, time of day and number of problems). NTAA was given more frequently in the lower age groups, during the night, and when the number of presented complaints was less than three. Finally, the proportions of presented problems that were handled through NTAA ranged from 5.4% for chest pain to 47.9% for earache.

**Table 1:** Number (valid %) of calls handled by the nurse alone and subsequent (first) return consultations

	Total number of calls	Handled by nurse alone	
		Initial triage	First return consultation*
	n (%)	n (%)	n (%)
Sex			
Male	659 (46.4)	180 (27.3)	66/179 (36.9)
Female	762 (53.6)	211 (27.7)	66/211 (31.3)
			
Age group (yrs)			
0–4	444 (31.3)	148 (33.3)	56/148 (37.8)
5–14	211 (14.9)	67 (31.8)	20/67 (29.9)
15–24	99 (7.0)	32 (32.3)	10/32 (31.3)
25–44	256 (18.0)	67 (26.2)	16/67 (23.9)
45–64	179 (12.6)	34 (19.0)	14/34 (41.2)
>65	231 (16.3)	43 (18.6)	16/42 (38.1)
			
Time of day			
Day (8 am-5 pm)	515 (36.6)	120 (23.3)	37/120 (30.8)
Evening (5 pm-11 pm)	641 (45.5)	154 (24.0)	46/154 (29.9)
Night (11 pm-8 am)	252 (17.9)	114 (45.2)	49/113 (43.4)
			
Number of problems			
1	334 (23.5)	103 (30.8)	38/103 (36.9)
2	598 (42.1)	184 (30.8)	61/184 (33.2)
3	489 (34.4)	104 (21.3)	33/103 (32.0)
			
Total	1421 (100.0)	391 (27.5)	132/390 (33.8)

* Percentage of initial triage, excluding patients who were referred to the hospital during their first out-of-hours contact

The group of eight nurses had a mean 21 years (range 13–27) of clinical experience. While three nurses had worked in more than three specialities, three had previously worked in a GP surgery.

Table 2 shows the initial set of variables and those that were retained in the final regression model. Nightly calls, earache, and vomiting were positively associated with NTAA (RR >1). Increasing age, >2 problems presented, chest pain, localised abdominal pain and shortness of breath were negatively associated with NTAA (RR <1). No associations were found with sex, type of insurance, social deprivation, or distance to the GP cooperative, or nurses' sex or prior clinical experience (20 years or more (y/n), more than three specialities (y/n), experience in GP practice (y/n)).

**Table 2:** Relative risks (RR) for determinants of nurse telephone consultation alone (NTAA) and return consultations after NTAA or after GP contact. Univariable and multivariable associations (95% CI).

	**Nurse telephone advice alone (NTAA)**		**Return consultations**		
			After NTAA	After NTAA	After GP contact
	Univariable associations	Multivariable model	Univariable associations	Multivariable model	Multivariable model
**Patient characteristics**	RR (95%CI)	RR (95%CI)	RR (95%CI)	RR (95%CI)	RR (95%CI)
Male	1.00 (ref)		1.00 (ref)		
Female	1.00 (0.84–1.19)	-	0.85 (0.61–1.14)	-	-
					
Public insurance	1.00 (ref)		1.00 (ref)		
Private insurance	1.02 (0.84–1.22)	-	1.01 (0.73–1.34)	-	-
					
Non-deprived area	1.00 (ref)		1.00 (ref)		
Deprived area	0.90 (0.75–1.08)	-	0.88 (0.64–1.17)	-	-
					
Distance per 5 km*	1.03 (0.78–1.31)	-	1.38 (0.94–1.83)	-	-
					
Age per 10 yrs**	0.92 (0.90–0.95)	0.96 (0.93–0.99)	0.99 (0.94–1.05)	1.02 (0.99–1.05)	1.02 (1.00–1.04)
					
Day or evening	1.00 (ref)	1.00 (ref)	1.00 (ref)	1.00 (ref)	1.00 (ref)
Night	1.52 (1.37–1.65)	1.63 (1.48–1.76)	1.33 (1.07–1.58)	1.23 (1.04–1.40)	1.28 (1.13–1.41)
					
1 or 2 problems presented	1.00 (ref)	1.00 (ref)	1.00 (ref)	1.00 (ref)	1.00 (ref)
3 problems presented	0.65 (0.52–0.82)	0.65 (0.51–0.83)	0.91 (0.63–1.25)	1.01 (0.82–1.16)	0.95 (0.86–1.03)
					
Cough	1.00 (ref)	1.00 (ref)	1.00 (ref)	1.00 (ref)	1.00 (ref)
Chest pain	0.17 (0.07–0.45)	0.18 (0.06–0.47)	#	#	0.81 (0.56–1.00)
Localised abdominal pain	0.36 (0.17–0.75)	0.35 (0.16–0.74)	1.22 (0.74–1.43)	#	1.50 (1.18–1.74)
Shortness of breath	0.44 (0.25–0.76)	0.41 (0.22–0.74)	1.21 (0.79–1.46)	1.21 (0.58–1.88)	0.90 (0.71–1.06)
Generalised abdominal pain	0.92 (0.58–1.39)	0.86 (0.53–1.32)	0.23 (0.07–0.70)	0.62 (0.29–0.92)	1.34 (1.09–1.53)
Sore throat	1.17 (0.80–1.62)	1.34 (0.92–1.82)	0.83 (0.41–1.38)	0.91 (0.55–1.24)	0.95 (0.77–1.09)
Fever	1.22 (0.89–1.59)	1.17 (0.83–1.56)	0.92 (0.54–1.36)	1.00 (0.66–1.31)	1.06 (0.90–1.17)
Headache	1.33 (0.96–1.73)	1.40 (1.00–1.81)	0.22 (0.08–0.64)	0.61 (0.31–0.90)	0.80 (0.56–0.96)
Vomiting	1.39 (1.10–1.68)	1.44 (1.20–1.63)	0.25 (0.11–0.54)	0.62 (0.39–0.85)	0.90 (0.73–1.02)
Earache	1.51 (1.30–1.68)	1.49 (1.18–1.78)	0.93 (0.56–1.36)	0.99 (0.69–1.27)	0.94 (0.75–1.07)
					
**Nurse-related variables**					
Male	1.00 (ref)		1.00 (ref)		
Female	1.03 (0.73–1.38)	-	1.18 (0.87–1.51)	-	n.a.
					
Experience < 20 years	1.00 (ref)		1.00 (ref)		
Experience > 20 years	1.02 (0.79–1.29)	-	0.86 (0.59–1.19)	-	n.a.
					
No. of specialities ≤ 3	1.00 (ref)		1.00 (ref)		
No. of specialities > 3	0.97 (0.73–1.25)	-	1.23 (0.95–1.51)	-	n.a.
					
No experience in GP practice	1.00 (ref)		1.00 (ref)		
Experience in GP practice	0.98 (0.70–1.32)	-	1.29 (1.03–1.55)	-	n.a.

GP = general practitioner; ref = reference category; * distance to GP cooperative, reference 0–1 kilometre; ** reference 0 years; age and distance were modelled as continuous variables; # numbers too low for analysis; n.a. = not applicable

**Figure 2:**
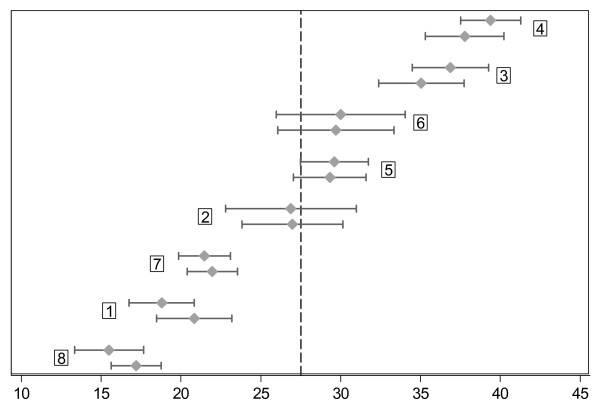
**Variability in the percentage of calls dealt with by nurse telephone advice alone (NTAA rate) among eight nurses working in a Dutch GP cooperative.** NTAA rates (dots) and their 95% confidence limits (lines) for each of eight nurses. The upper lines indicate the unadjusted NTAA rates for each nurse, whereas the bottom lines indicate the rates adjusted for age, time of contact, and number and type of presented problems. Note that the adjustment brings the rates closer to the overall mean (27.5%, dotted vertical line). The numbers in the boxes represent the nurse identification numbers.

The median number of contacts per nurse was 188 (IQR 147 to 301). The average percentage of NTAAs across all presented problems ranged from 15.5% to 39.4% for the eight nurses (Figure 2). This amount of between-nurse variability can also be expressed as the intra-class correlation (ICC), that is, the percentage of all variation in the NTAA rates that is due to differences between the nurses. After fitting the most parsimonious model, the proportions of calls with NTAA showed significant variability between the nurses (p < 0.001) justifying random-effects analysis. Figure 2 shows how this adjustment brings the individual NTAA rates closer to the overall mean (27.5%), although considerable, unexplained inter-nurse variability remains.

### Return consultations: determinants and hospital referrals

After NTAA, 33.8% (132/390) of the patients returned to the GP within the first two weeks of the out-of-hours contact (Fig. 1). A nightly contact was positively associated with a return consultation, while headache and vomiting were negatively associated with a return consultation after NTAA (Table 2). Again, return consultation rates were not found to be associated with sex, insurance type, social deprivation, travelling distance to the GP cooperative, or nurses' sex or prior clinical experience. Since the number of 132 (out of 390) patients returning after NTAA was too low to allow complete adjustment for case-mix differences (being divided across 8 (nurses) times 10 (types of problems)), no variability could be detected between the nurses with regard to the proportions of return consultations.

The return consultation rate for patients who had had out-of-hours contact with the GP was 26.9% (250/928)(Fig. 1). Determinants of return consultations after these GP contacts are presented in Table 2 for global comparisons only. Interestingly, a nightly contact was also associated with a higher return consultation rate, while general abdominal pain showed the clearest differences between NTAA and GP consultation.

Finally, the median time to first return consultation appeared to be shorter after NTAA than after a GP contact: one and two days, respectively (log rank test p = 0.0041). Also, during the first return consultation with the GP, patients who had received NTAA were less often referred to the hospital than those who had initially come into contact with a GP: 1.5% (6/390) versus 3.8% (35/928) (difference -2.2%; 95% CI = -4.0 to -0.5%). Overall, patients who contacted the GP cooperative during the night were more likely to be referred to the hospital than during the day or evening (10.3% (26/252) versus 6.5% (75/1156); difference 3.8%; 95% CI = -0.2 to 7.8%).

## Discussion

In this study, various determinants of NTAA and return consultations were found. Telephone nurses appeared most confident in providing advice to parents of young children and in addressing problems like earache, vomiting, and cough while they were more cautious when more than two problems were presented or when the presented problem involved chest pain, localised abdominal pain or shortness of breath. During the night, the nurses were more likely to provide NTAA compared to the day or evening. After correction for these factors, significant variability among the eight nurses remained. The probability of return consultations appeared to be associated mainly with after midnight calls and the type of presented problem. The patients who were referred to the GP by the nurse were more likely to be referred to the hospital, both during their first out-of-hours contact and first return consultation.

The GP cooperative studied had the advantage of an unequivocal accessibility of electronic medical records for both in- and out-of-hours consultations. However, one needs to bear in mind that the cooperative studied was somewhat different from most others as it was located in a former A&E Department rather than a primary care centre and had employed former A&E nurses rather than practice nurses. Moreover, since the number of nurses who participated in this study was rather small, the results may not be generalisable to other GP cooperatives. Nevertheless, there are many similarities between our results and those from another Dutch study regarding overall demand and NTAA rate [19], which increases the likelihood that the results from both studies may be applicable to other areas of the country.

Another limitation of the study is that the collection of follow-up data took place more than two years after the initial data collection. Fortunately, since the GPs keep their electronic medical records (EMR) for a period of at least ten years after patients have died or moved elsewhere, the number of missing data remained very limited. Compared to the EMR, there appeared to be a general underestimation of the prospectively registered contacts of 2.1% (data not shown; mainly contacts concerning repeat prescriptions), indicating that the overall reliability of the first contact data is satisfactory. However, GPs or practice nurses may not always have entered information in the EMR when patients re-contacted their surgery in the daytime, although we believe that the financial incentive to claim all surgery contacts will have limited the number of missing data.

In this study, no quantification could be given for differences in severity of illness within the types of studied problems, leaving some room for residual confounding. Furthermore, the higher onward hospital referral rate after GP contacts compared to NTAA may indicate a higher level of complexity, but this association is perhaps confounded by the GPs' cautiousness and higher propensity to refer patients to the hospital who revisit their surgery after an out-of-hours contact with a fellow GP.

Finally, the small number of nurses in this study allowed for the inclusion of only a few nurse-related characteristics, such as length of clinical experience (both in- and outside the GP practice) or variety of experience [15]. Nevertheless, in addition to being important in explaining differences in clinical behaviour between nurses (as illustrated by NTAA), these or similar variables may also be amenable to modification through continuing education. Further research including larger numbers of nurses is needed to explore the effect of nurse-related features on the provision of telephone advice.

Although various studies have described the process of telephone triage in out-of-hours primary care services [5,7,8], factors related to the NTAA process or the extent of inter-nurse variability has, in our opinion, received little attention [11,15,20]. Nevertheless, while inter-nurse variability may indicate fields of disagreement on task definition among nurses, determinants of NTAA could also contribute to defining its domain.

Studies from the UK have indicated that through the use of telephone advice systems (TAS) [6], telephone nurses can safely handle up to 50% (or more) of the incoming calls [5,7,8]. This suggests that the use of such systems may facilitate a substantial increase in Dutch NTAA rates, although its effects on inter-nurse variability [15] and return consultations have yet to be established. As Wachter et al. have also pointed out, it should not simply be assumed that (intensified) use of telephone triage protocols will standardise care and the consistency of these protocols needs to be validated before safe dissemination for general use can take place [21].

We found that nurses handled a larger proportion of calls alone at night than during the day and evening. While after midnight calls are thought to be of a more serious nature [22], as is perhaps supported by our finding that more patients were referred to the hospital during the night than during the day and evening, we would have expected the NTAA rate to go down during the night. At least two mechanisms may, in combination, be responsible for this finding: explicit instruction to triage more strictly or implicit perception of a higher threshold to consult the GP who may have been asleep or out on a visit. More research is needed to answer the question whether nurses take on more complex cases during nightly calls and to study to what extent this affects the quality and safety of care [23].

In this study we found that 33.8% of the patients returned for a consultation with the GP after NTAA, with a median return time of one day. Interestingly, only 26.9% of the patients returned following a GP contact (difference 6.9%; CI 1.4–12.4), after a median period of two days. If the nurses handle the more straightforward, simple cases and refer the more complex cases to the GP (as is supported by the differences in hospital referral rates during first out-of-hours contact and first return consultation), it may seem counter-intuitive that the patients return both earlier and more often to the GP after talking to the telephone nurse. Perhaps this reflects that nurses distinguish between problems that need immediate attention and problems that can wait until the surgery hours, thereby referring some of the patients back to their own GP as has been suggested before. On the other hand, this may also have been the result of a lower confidence in, or reassurance by, the telephone nurses [24-26] or some degree of discontent due to a mismatch between the care expected (e.g. a home visit) and the care received (telephone advice) [27,28].

Given the large variation in independent NTAA rates in the literature, the professional role of telephone nurses needs to be further defined. In this process, a more comprehensive use of telephone advice systems may increase NTAA rates and decrease inter-nurse variability, resulting in a higher overall effectiveness of nurse telephone triage [15,29]. Nevertheless, no matter what decision support systems nurses may rely on, telephone triage appears to be a very complex procedure that requires specific skills [30-32]. These skills should become part of ongoing educational training programs that make nurses more aware of their professional role and boundaries [33,34], the limitations imposed by lack of visual cues [35], and the strengths and limitations of their decision support systems [36]. Perhaps more attention should also be paid to issues like reassurance [24], care expectancy [27], or the possibility to talk to a doctor (like telephone doctors) [28]. In doing so, training may lead to higher levels of confidence and a more positive attitude [37] and, ultimately, to a higher quality and safety of telephone triage and consultation [38,39].

## Conclusion

In this study, various determinants of NTAA and return consultations were found. However, important inter-nurse variability may indicate differences in perception on tasks and/or differences in skill to handle telephone calls alone. Further discussion is needed to define the optimal role of the nurse in the telephone triage while future research should focus more on modifiable determinants of NTAA rates.

## Competing interests

The author(s) declare that they have no competing interests.

## Authors' contributions

EMC planned the design of the study, collected the first contact data, took part in the analysis and led the writing of the paper. GR analysed the data, made substantial contributions to the figures and tables and was involved in drafting the manuscript. SD and LL collected the data and were involved in drafting the paper. NSK took part in drafting the paper. PB made substantial contributions to the study design and interpretation of data as well as to drafting the paper. All authors read and approved the final version of the manuscript.

## Pre-publication history

The pre-publication history for this paper can be accessed here:


